# Elastic deformation of twinned microstructures

**DOI:** 10.1098/rspa.2017.0330

**Published:** 2017-08-16

**Authors:** Steffen Pfeiffer, Martin Franz-Xaver Wagner

**Affiliations:** Institute of Materials Science and Engineering, Chemnitz University of Technology, Chemnitz, Germany

**Keywords:** twins, elastic anisotropy, compatibility stresses, NiTi shape memory alloys, Cu twins

## Abstract

Many crystalline materials exhibit twinned microstructures, where well-defined orientation relationships define the special symmetry between different, elastically anisotropic twin variants. When such twins are subjected to external loading, additional internal stresses necessarily occur at the twin boundaries in order to maintain compatibility. These compatibility stresses are constant inside each variant in repeating stacks of twins and considerably affect the local stress state. In this paper, we use anisotropic linear elasticity to derive general analytical solutions for compatibility stresses in a stack of twin variants in arbitrary materials, for arbitrary variant volume fractions and twin types, subjected to arbitrary applied stresses. By considering two examples, growth twins in electrodeposited Cu and B19′ martensite twins in the shape memory alloy NiTi, we further demonstrate that compatibility stresses can considerably alter the preferred slip systems for dislocation plasticity as well as the effective macroscopic behaviour of twinned microstructures.

## 1. Introduction

When two materials are joined at an interface and subjected to external mechanical loading, even in the simplest case of purely elastic deformation, additional stresses at the interface are required in order to maintain compatibility. This phenomenon has been particularly recognized by scientists studying fatigue and fracture of metals and alloys, where compatibility stresses at internal boundaries are known to determine crack initiation sites and crack growth [1,2]. The underlying, quite fundamental question in many related situations is: How does elastic anisotropy affect the stress state at interfaces in materials that are subjected to external loads?

Polycrystalline materials can exhibit many different types of interfaces (for instance, small and large angle grain boundaries, phase boundaries or twin boundaries), with various degrees of freedom, three-dimensional curvature and numerous topological characteristics on the atomistic length scale, such as steps and ledges. A detailed model of material interfaces also often requires to explicitly include interfacial defects like dislocations or disconnections, which can be described as a generalized type of disclinations [3]. Devising an accurate, all-encompassing description of the many properties and features of interfaces, and of the complex stress states in their vicinity once an oligo- or polycrystalline specimen is subjected to external loading, certainly is a formidable task.

Early research in materials science and mechanics has often been focused on modelling special cases, such as the stress fields surrounding ellipsoidal inclusions [4,5], or at special types of grain boundaries [6]. Inclusion problems in particular have been the object of numerous studies, as summarized for instance in [7]. Theoretical and experimental studies on anisotropic stresses and their effect on the interaction of dislocations with grain boundaries [8–10] or twin boundaries [11,12] have been performed. Mechanical testing on bicrystals containing single grain or twin boundaries has been used to investigate the activation of slip systems, plastic deformation and fracture near the interfaces, using both macroscopic and, in recent years, micro-scale samples [10,11]. The effect of elastic and plastic anisotropies has also been considered in theoretical and numerical studies on stress and strain distributions near interfaces and on their impact on the macroscopic material behaviour, for instance using field dislocation mechanics [13], crystal plasticity models [14] or atomistic calculations [15]. While these modelling approaches provide detailed insights into relevant microstructural deformation mechanisms, they are expensive in terms of computing power and time, and they are usually limited to specific types of materials, interfaces and load cases.

**Figure 1.**
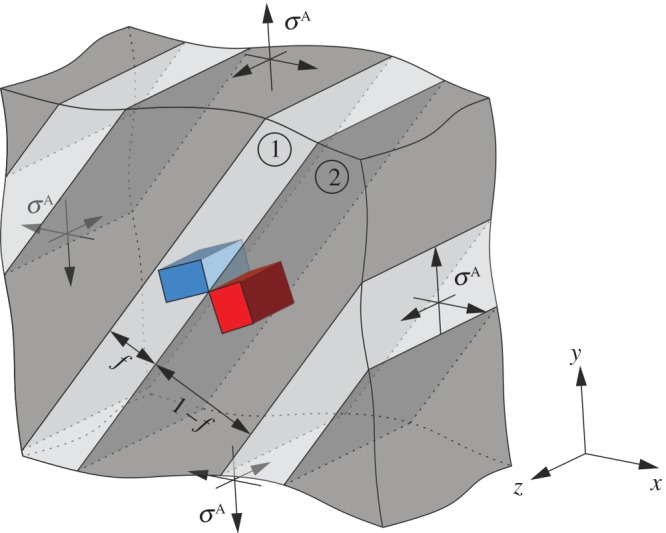
Schematic of a twinned microstructure subjected to external loading. Two variants (1 and 2; characterized by the orientation of the corresponding crystal lattices as indicated by their blue/red unit cells) and volume fractions, *f* and (1 − *f*), respectively, form the twin. A special orientation relationship exists between the two variants, and we consider a regular stack of the variant pair to form a representative twinned microstructure. (Online version in colour.)

A simple, yet very general, analytical model of elastic compatibility stresses can, however, be formulated for an important subset of material interfaces: twin boundaries. Twins and twin boundaries are prominent microstructural elements in many materials, determining both structural and functional properties in, e.g. ultra-high strength steels, lightweight magnesium and titanium alloys or in smart materials like shape memory alloys. As shown schematically in figure 1, we consider a twinned microstructure as a regular ‘stack’ of repeating layers, i.e. of two twin variants. This type of sequential laminate is also known as polysynthetic twin in mineralogy [16]; such structures can also be considered as representative for twinned volumes in the individual grains of a polycrystal. In taking this view, we neglect the experimental observation that twins typically exhibit a lenticular shape by becoming thinner in the vicinity of obstacles like grain boundaries. This effect is related to additional geometrical constraints and to a tendency to reduce elastic strain energy during twin formation [17] and is not further considered here.

There are well-defined orientation relationships between the crystal lattices of the twin variants (shown in figure 1 by the different orientations of their unit cells), which are typically known from experiment or which can be determined from theoretical calculations [17] for specific crystal lattices. Most importantly, a twin boundary separating two crystallographic variants in the absence of external loading is a planar and unstrained interface of very low energy that contains little or no interfacial defects. Because of the simplicity of these microstructures, the high degree of symmetry and the known orientation relationships between individual twin variants, twin boundaries have been recognized as an ideal model system to study compatibility stresses. Earlier studies have shown that the compatibility stresses in twinned microstructures can be of the same order of magnitude (and in special cases higher by a factor of up to three) as externally applied stresses [18–22]. A general model of compatibility stresses in twinned microstructures promises relevant information and new insights for a wide range of materials.

In this paper, we develop a simple analytical model of compatibility stresses in twinned microstructures by extending an approach that has been originally proposed for bicrystals [23]. While the original model presented in [23] considers the general case of two arbitrary, elastically anisotropic materials joined at a planar interface, we incorporate the special symmetry related to twinned structures to derive a model specifically valid for different types of twins and simpler to use. The model allows to directly calculate compatibility stresses from the applied stress state for arbitrary types of twins and materials. We, moreover, demonstrate that compatibility stresses are constant throughout individual variants; consequently, they can have a profound effect on the elastic stress fields, on microstructural deformation mechanisms and on the resulting macroscopic material behaviour of twinned materials. To further investigate some interesting properties of our model, we study two examples that are of special relevance for materials science: (i) we discuss how compatibility stresses affect the activation of dislocation slip systems for plastic deformation in twinned copper and (ii) we analyse the effect of compatibility stresses on the macroscopic elastic properties and on elastic strain energy of twinned B19′ martensite in the shape memory alloy NiTi.

## 2. Symmetry relations for the elastic properties of different twin types

**Figure 2.**
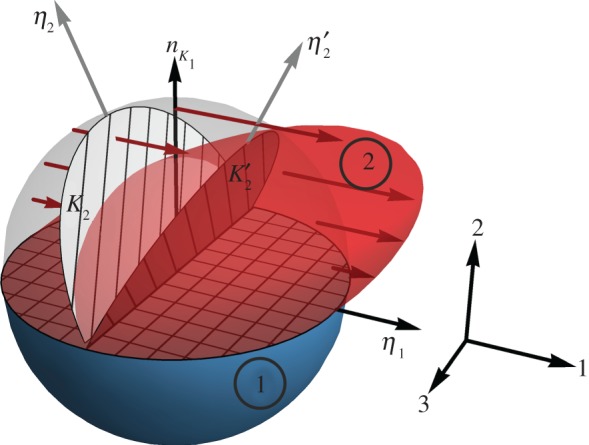
Schematic representation of a unit sphere with all relevant crystallographic parameters to define the twinning elements. The lattice of variant 2 (upper half) is formed by shearing the lattice of variant 1. *η*_1_ is the shear direction, contained within the twinning plane *K*_1_. Mechanical properties of the twinned material can be described using the Cartesian coordinate system with directions 1, 2 and 3. (Online version in colour.)

In this section, we define the fundamental geometrical relations to describe a twin, and we analyse how the special symmetry relating twin variants in different types of twins can be used to describe the elastic properties in a global coordinate system. We first recall the common definition of the so-called twinning elements used in crystallography and physical metallurgy to describe the (purely geometrical) orientation relationship between the crystal lattices of the two crystallographic variants [24]. Directions are defined relative to the (usually non-Cartesian) coordinate system tied to the lattice vectors of the relevant crystal structure for the material under consideration, and planes are defined using Miller indices in the same coordinate system. As shown schematically in figure 2 for a unit sphere, we consider the planes *K*_1_ and *K*_2_, and the directions *η*_1_ and *η*_2_, defined in the lattice of the first variant. A simple shear operation (amount of shear: *s*) on the upper half of a unit sphere transforms the lattice of variant 1 into the lattice of variant 2. The undistorted plane containing the direction of shear, *η*_1_, is known as the twin boundary or twinning plane *K*_1_ (orientation given in Miller indices). It separates the undeformed, lower half of the unit sphere (variant 1) from the shear-transformed, upper half (variant 2). A second plane, *K*_2_, and the direction *η*_2_ contained therein, also remain undistorted during the shear operation; they are simply rotated to become $K_{2}^{\prime }$ and $\eta_{2}^{\prime }$ in the lattice of variant 2. It is common in physical metallurgy to distinguish twins based on the microstructural mechanism responsible for their formation. Only mechanical twinning (deformation twinning) is physically associated with a shear process as shown in figure 2. Transformation twins (martensite twins) are formed during a diffusion-less phase transformation from a parent phase; while this phase transformation also involves a shear deformation to transform the parent phase lattice into the twinned lattices of martensite variants, the shear direction typically differs from *η*_1_ used to describe the geometry of the martensite twins themselves. Finally, growth twins result from individual atomic movements in diffusive processes, e.g. during solidification from a melt, annealing of a solid or deposition on a substrate from the vapour phase [25]. Irrespective of the formation mechanism (and irrespective of the twin type as defined below), the orientation relationship between the twin variants can always be completely defined using the crystallographic twinning elements.

Figure 2 also shows a Cartesian coordinate system that is particularly useful to develop a model of the elastic behaviour of the twinned microstructure, with the 1-direction pointing along the shear direction *η*_1_, the 2-direction normal to the twin plane and the 3-direction contained therein. This Cartesian coordinate system is used consistently for all theoretical considerations below, and referred to as *twin coordinate system*. Throughout this paper, we primarily use Voigt notation instead of tensor notation; Hooke's Law for anisotropic, linear elasticity is then given by

2.1 $$\sigma_{i}=C_{ij}\varepsilon_{j}$$

or by the inverse relation

2.2 $$\varepsilon_{i}=S_{ij}\sigma_{j},$$

where the components of the stress and strain tensors are rearranged into 6 × 1 vectors *σ_i_* and ε*_i_*, and *C_ij_* and *S_ij_* are the 6 × 6 stiffness matrix and its inverse, the compliance matrix, respectively. Because of the symmetry of stress and strain tensors, the *C_ij_* and *S_ij_* matrices are also symmetric; they can contain a maximum of 21 independent components (*c_ij_* = *c_ji_* or *s_ij_* = *s_ji_*)—typically much less because of crystal symmetry, when the elastic properties of a single crystal are described in a Cartesian coordinate system that is oriented properly with respect to the symmetry planes of the crystal (e.g. defined in [26] for the seven crystal systems). Typically, this material-fixed coordinate system is different from the twin coordinate system, and a coordinate transformation is needed to describe the elastic properties of each variant in the twin coordinate system. While this is generally more easily performed in tensor notation, we highlight that the matrix manipulation procedure outlined e.g. in [27] can be applied to avoid the tiresome conversion between Voigt and tensor notations. Note that even when considering high-symmetry crystal structures, where the number of independent elastic constants is considerably reduced compared with the triclinic case (e.g. in the case of cubic symmetry, only *c*_11_ = *c*_22_ = *c*_33_ ≠ 0, *c*_12_ = *c*_13_ = *c*_23_ ≠ 0 and *c*_44_ = *c*_55_ = *c*_66_ ≠ 0), the *C_ij_* or *S_ij_* matrices typically contain considerably more non-zero components when described in the twin coordinate system. In the most general case of 21 independent, non-zero components when *C_ij_* is described in the twin coordinate system, the stiffness matrix of variant 1 is given by

2.3 $${}^{1}C_{ij}=\left(\begin{array}{cccccc} c_{11} & c_{12} & c_{13} & c_{14} & c_{15} & c_{16}\\ & c_{22} & c_{23} & c_{24} & c_{25} & c_{26}\\ & & c_{33} & c_{34} & c_{35} & c_{36}\\ & & & c_{44} & c_{45} & c_{46}\\ & \text{sym}. & & & c_{55} & c_{56}\\ & & & & & c_{66} \end{array}\right).$$

**Figure 3.**
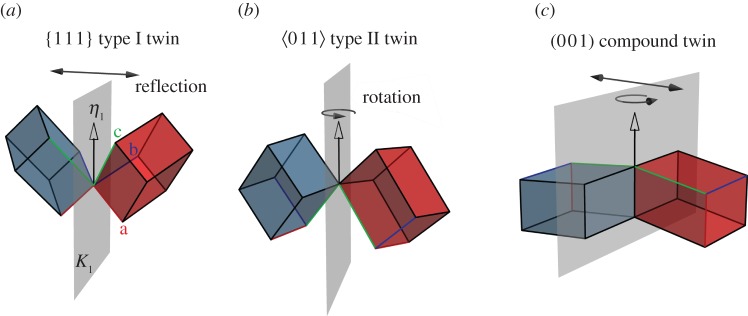
The three twin types can be illustrated considering different twins (all of them observed experimentally) in monoclinic B19′, the martensitic phase of the shape memory alloy NiTi. The twin plane is represented in grey and the *η*_1_-direction is indicated by an open arrow; the unit cell of variant 1 (blue) is transformed into the unit cell of variant 2 (red) by different operations: (*a*) a {1 1 1} type I twin is formed by a mirror operation, where the {1 1 1} plane of variant 1 is parallel to the twin plane; (*b*) a ⟨0 1 1⟩ type II twin is associated with a rotation of the unit cell by 180° around the ⟨0 1 1⟩ direction; (*c*) a (0 0 1) compound twin exhibits both a mirror symmetry with respect to the (0 0 1) plane and rotational symmetry with respect to the [1 0 0] direction. (Online version in colour.)

To conveniently develop our model of elastically anisotropic behaviour of twinned microstructures, we need to distinguish three types of twins based on the symmetry relation between the unit cells of variants 1 and 2. One example of every twin type is shown schematically in figure 3, using as an example the monoclinic B19′ unit cells that are observed in the shape memory alloy NiTi (see also §5): In type I twins (figure 3*a*), the unit cells of variants 1 and 2 are related by a mirror symmetry. The twin plane corresponds to the plane of reflection. Type II twins (figure 3*b*), by contrast, exhibit a rotational symmetry; the unit cell of variant 2 is produced by rotating the lattice of variant 1 by 180° around *η*_1_. Compound twins are characterized by the highest degree of symmetry, exhibiting both mirror and rotational symmetries.

For clarity, we use the following notation with regard to vectors and matrices in the remainder of this paper: superscripts on the left-hand side of a variable indicate the variant (1 or 2) under consideration; subscripts on the left-hand side indicate the twin type (I, II or C); superscripts on the right-hand side denote whether a variable is related to applied, compatibility or effective stresses (A, C or eff); subscripts on the right-hand side contain integers (e.g. *i*, *j*, *k*) to indicate the position of individual components. These subscripts and superscripts are only completely given where necessary.

The special symmetry of twinned microstructures allows to directly determine the components of the stiffness or compliance matrices of variant 2 once those of variant 1 are known (both described in the twin coordinate system). Consider a matrix ^1^*X* that contains either the *c_ij_* values of variant 1 (as given in equation (2.3)), or its *s_ij_* values. In the case of type I twins, the unit cell of variant 2 is formed as a mirror image of variant 1 (figure 3*a*); its elastic properties, therefore, are described by the same matrix—but in a coordinate system that is a mirrored (and, therefore, left-handed) version of the twin coordinate system. The unit vectors of this modified coordinate system, as seen from the twin coordinate system, therefore, are (1,0,0), (0,−1,0), (0,0,1). Performing a coordinate transformation from the mirrored coordinate system to a description of ^2^*X* in the twin coordinate system is straightforward, and by comparing the components of ^1^*X* and ^2^*X*, we find that individual components *x_ij_* either have the same value in both matrices, or they have the same absolute value, but different signs. This result is summarized by

2.4 $${}_{I}X=\left(\begin{array}{cccccc} • & • & • & \circ & • & \circ \\ & • & • & \circ & • & \circ \\ & & • & \circ & • & \circ \\ & & & • & \circ & •\\ & \text{sym}. & & & • & \circ \\ & & & & & • \end{array}\right)\;\begin{array}{cc} • & \text{const}.\\ \circ & \text{CS} \end{array},$$

where a dot indicates that a component has the same value in both variants, and an open circle represents a component with a change of sign (CS) when one compares variants 1 and 2. We highlight that this result is generally valid for type I twins for all possible crystal systems.

In the case of type II twins, the unit cell of variant 2 is obtained by rotation (by 180° around *η*_1_, figure 3*b*) of the unit cell of variant 1; when the elastic properties of variant 2 are described in a similarly rotated coordinate system, its stiffness (or compliance) matrix is identical to that of variant 1 as described in the twin coordinate system. The rotated coordinate system is characterized by unit vectors (1,0,0), (0,−1,0), (0,0,−1). Transforming (the matrix ^2^*X*) back towards the twin coordinate system and comparing individual components, one can easily show that also for type II twins most components *x_ij_* have the same values in both variants, and only some components have the same values, but a different sign:

2.5 $${}_{\mathrm{II}}X=\left(\begin{array}{cccccc} • & • & • & • & \circ & \circ \\ & • & • & • & \circ & \circ \\ & & • & • & \circ & \circ \\ & & & • & \circ & \circ \\ & \text{sym}. & & & • & •\\ & & & & & • \end{array}\right)\begin{array}{cc} • & \text{const}.\\ \circ & \text{CS} \end{array}.$$

Based on these symmetry relationships, one can formally calculate all elastic constants of variant 2 (and, similarly, all compliance constants) from those of variant 1 using the transformation rule

2.6 $${\;}^{2}C_{ij}=K_{ij}\cdot^{1}C_{ij}\cdot K_{ij}.$$

This transformation rule represents a special case of the general expression for coordinate transformations described in [27] and has been simplified using the fact that only diagonal elements of *K_ij_* are non-zero. Equation (2.6) is equivalent to the operations described in equations (2.4) and (2.5), but more convenient for some of the matrix analysis needed in subsequent sections. For type I twins, the corresponding transformation matrix is

2.7 $${}_{I}K=\left(\begin{array}{cccccc} 1 & 0 & 0 & 0 & 0 & 0\\ 0 & 1 & 0 & 0 & 0 & 0\\ 0 & 0 & 1 & 0 & 0 & 0\\ 0 & 0 & 0 & -1 & 0 & 0\\ 0 & 0 & 0 & 0 & 1 & 0\\ 0 & 0 & 0 & 0 & 0 & -1 \end{array}\right),$$

whereas for type II twins equation (2.6) needs to be used in combination with

2.8 $${}_{\mathrm{II}}K=\left(\begin{array}{cccccc} 1 & 0 & 0 & 0 & 0 & 0\\ 0 & 1 & 0 & 0 & 0 & 0\\ 0 & 0 & 1 & 0 & 0 & 0\\ 0 & 0 & 0 & 1 & 0 & 0\\ 0 & 0 & 0 & 0 & -1 & 0\\ 0 & 0 & 0 & 0 & 0 & -1 \end{array}\right).$$

As introduced above, compound twins (figure 3*c*) are characterized both by rotational and mirror symmetry. Obviously, both equations (2.4) and (2.5) must be valid (similarly, one can use either _I_*K* or _II_*K* when describing the elastic symmetry of a compound twin)—but computations must lead to the same result. This condition can only be fulfilled when the eight components that are characterized by different symbols in equations (2.4) and (2.5) are zero:

2.9 $$x_{14}=x_{24}=x_{34}=x_{15}=x_{25}=x_{35}=x_{46}=x_{56}=0.$$

We note in passing that this represents a direct and simple proof that only crystal structures with monoclinic symmetry (as characterized by 13 independent elastic constants) or with a higher degree of symmetry (i.e. a smaller number of independent elastic constants) can form compound twins, whereas triclinic crystals cannot, which has previously been rationalized using symmetry arguments [28].

## 3. General solutions for compatibility stresses in twins subjected to external loading

**Figure 4.**
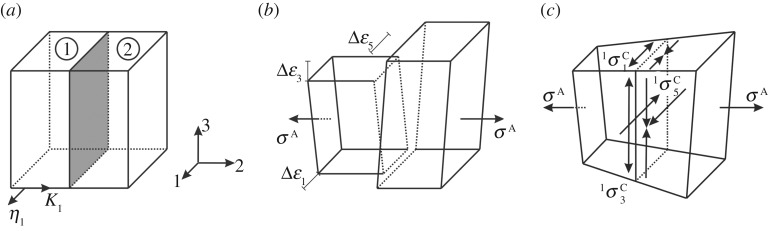
Formation of compatibility stresses at the interface of a bicrystal under external loading (schematic figure modified and adapted from [29]). (*a*) Interface of two crystals (e.g. twin variants) 1 and 2 in the unloaded condition, and relative orientation of twinning elements *K*_1_ and *η*_1_ for the special case of a twin boundary. (*b*) Free deformation of the two crystals due to their orientation and elastic anisotropy under external loading leads to strain incompatibility at the interface. (*c*) Compatibility at the interface requires that additional compatibility stresses (with different signs on both sides of the interface) occur.

To analyse the stress state at a single twin boundary, we closely follow the approach of Gemperlová *et al*. [23] for bicrystals, as summarized by Sutton & Balluffi [29]. Consider two materials (variants) 1 and 2 separated by a plane interface (twin boundary), as shown schematically in figure 4*a*. Both materials are characterized by anisotropic elastic properties. This ‘bi-material specimen’ is subjected to homogeneous, external loading (applied stress: $\sigma_{i}^{A}$). Without coherence at the twin boundary, both variants would deform differently (figure 4*b*). The resulting differences in terms of three strains (calculated using Hooke's Law, equation (2.2), the compliance matrices of both variants, and using the summation convention with *j* = 1 to 6)

3.1 $$\Delta \varepsilon_{k}={}^{2}s_{kj}\;\sigma_{j}^{A}-{}^{1}s_{kj}\;\sigma_{j}^{A}\;(k=1,3,5),$$

are, however, not permissible because free deformation would violate compatibility at the interface (note that the other three strain components may differ in both variants). Additional compatibility stresses $\sigma_{k}^{C}$ (with non-zero components for *k* = 1,3,5), therefore, must occur on both sides of the interface, such that the additional strains introduced by these stresses counterbalance the Δε*_k_*. This leads to the condition

3.2 $${}^{2}s_{kj}(\sigma_{j}^{A}+{}_{}^{2}\sigma_{j}^{C})-{}^{1}s_{kj}(\sigma_{j}^{A}+{}^{1}\sigma_{j}^{C})=\Delta \varepsilon_{k}+{}^{2}s_{kj}{}^{2}\sigma_{j}^{C}-{}^{1}s_{kj}{}^{1}\sigma_{j}^{C}=0\;(k=1,3,5).$$

In the limiting case of a bicrystal consisting of two semi-infinite half-spaces (discussed by Gemperlová *et al*. [23]), compatibility stresses at the interface have the same magnitude, but different signs in both materials (a consequence of global stress equilibrium), and the three equations summarized in equation (3.2) can be solved numerically to provide the three compatibility stresses. In a finite-sized bicrystal sample they need to decay quickly with increasing distance from the single interface such that they vanish at the free surfaces [29].

Considering the present investigation, the model can also be applied to the case of a layered structure of repeating variants with volume fractions *f* and (1 − *f*), [23]. It is important to note that in this general case of a layered structure, because they must occur on both sides of each variant, the compatibility stresses also do not decrease with increasing distance from the twin boundary: they remain constant throughout each variant. In contrast to the situation studied originally in bicrystals, where the model only provides information on the stress state in the vicinity of a single interface, compatibility stresses in twinned structures are superimposed on the applied stress state in the entire volume. The modified, homogeneous stress state in twinned microstructures, therefore, can have a pronounced effect on microstructural deformation mechanisms and macroscopic (effective) material behaviour. Stress equilibrium in this case requires that

3.3 $$f\;{}^{1}\sigma_{k}^{C}+(1-f)\;{}^{2}\sigma_{k}^{C}=0\;(k=1,3,5).$$

Only when *f* = 0.5, compatibility stresses have the same absolute value in both variants. Generally, they have a higher absolute value in variants with a low volume fraction (*f* < 0.5) and lower absolute values in variants with a high volume fraction (*f* > 0.5).

Combining equations (3.2) and (3.3), we can eliminate the ${}_{}^{2}\sigma_{k}^{C}$ and thus obtain a set of three equations that can be solved numerically to calculate the three compatibility stresses in variant 1 for arbitrary external loading (as expressed by the left-hand side terms that are a function of the components of the applied stress state $\sigma_{i}^{A}$):

3.4 $$\begin{array}{rl} \Delta \varepsilon_{1} & ={}^{1}s_{11}{}^{1}\sigma_{1}^{C}+{}_{}^{1}s_{13}^{}{}_{}^{1}\sigma_{3}^{C}+{}_{}^{1}s_{15}^{}{}_{}^{1}\sigma_{5}^{C}+\frac{f}{1-f}\;({}_{}^{2}s_{11}^{}{}_{}^{1}\sigma_{1}^{C}+{}_{}^{2}s_{13}^{}{}_{}^{1}\sigma_{3}^{C}+{}_{}^{2}s_{15}^{}{}_{}^{1}\sigma_{5}^{C}), \end{array}$$

3.5 $$\begin{array}{rl} \Delta \varepsilon_{3} & ={}_{}^{1}s_{13}{}^{1}\sigma_{1}^{C}+{}_{}^{1}s_{33}^{}{}_{}^{1}\sigma_{3}^{C}+{}_{}^{1}s_{35}^{}{}_{}^{1}\sigma_{5}^{C}+\frac{f}{1-f}\;({}_{}^{2}s_{13}^{}{}_{}^{1}\sigma_{1}^{C}+{}_{}^{2}s_{33}^{}{}_{}^{1}\sigma_{3}^{C}+{}_{}^{2}s_{35}^{}{}_{}^{1}\sigma_{5}^{C}) \end{array}$$

3.6 $$\begin{array}{rl} \;\text{and}\;\Delta \varepsilon_{5} & ={}^{1}s_{15}^{}{}_{}^{1}\sigma_{1}^{C}+{}_{}^{1}s_{35}^{}{}_{}^{1}\sigma_{3}^{C}+{}_{}^{1}s_{55}^{}{}_{}^{1}\sigma_{5}^{C}+\frac{f}{1-f}\;({}_{}^{2}s_{15}^{}{}_{}^{1}\sigma_{1}^{C}+{}_{}^{2}s_{35}^{}{}_{}^{1}\sigma_{3}^{C}+{}_{}^{2}s_{55}^{}{}_{}^{1}\sigma_{5}^{C}). \end{array}$$

Note that both the right-hand sides and the left-hand sides of equations (3.4)–(3.6) contain components of the compliance matrices of both variants, ^1^*s_ij_* and ^2^*s_ij_*. We further take advantage of the special symmetry relations for different twin types to considerably simplify equations (3.4)–(3.6), and to analytically solve for the compatibility stresses in variant 1 as a function of applied stresses and using only components of the compliance matrix of variant 1. For type I twins, using equation (2.4), we obtain

3.7 $$\begin{array}{rl} {}_{I}\sigma_{1}^{C} & =\frac{2(-1+f)}{M}\{\sigma_{4}^{A}(-s_{15}s_{34}s_{35}+s_{14}s_{35}^{2}+s_{15}s_{33}s_{45}-s_{13}s_{35}s_{45}-s_{14}s_{33}s_{55}+s_{13}s_{34}s_{55})\\ & \;+\sigma_{6}^{A}(s_{16}s_{35}^{2}-s_{15}s_{35}s_{36}-s_{16}s_{33}s_{55}+s_{13}s_{36}s_{55}+s_{15}s_{33}s_{56}-s_{13}s_{35}s_{56})\}, \end{array}$$

3.8 $$\begin{array}{rl} {}_{I}\sigma_{3}^{C} & =\frac{2(-1+f)}{M}\{\sigma_{4}^{A}(-s_{14}s_{15}s_{35}+s_{15}^{2}s_{34}-s_{13}s_{15}s_{45}+s_{11}s_{35}s_{45}+s_{13}s_{14}s_{55}-s_{11}s_{34}s_{55})\\ & \;+\sigma_{6}^{A}(s_{15}^{2}s_{36}-s_{15}s_{16}s_{35}+s_{13}s_{16}s_{55}-s_{11}s_{36}s_{55}-s_{13}s_{15}s_{56}+s_{11}s_{35}s_{56})\} \end{array}$$

3.9 $$\begin{array}{rl} \;\text{and}\;{}_{I}\sigma_{5}^{C} & =\frac{2(-1+f)}{M}\{\sigma_{4}^{A}(s_{14}s_{15}s_{33}+s_{13}^{2}s_{45}-s_{13}s_{15}s_{34}-s_{13}s_{14}s_{35}+s_{11}s_{34}s_{35}-s_{11}s_{33}s_{45})\\ & \;+\sigma_{6}^{A}(s_{13}^{2}s_{56}+s_{15}s_{16}s_{33}-s_{13}s_{16}s_{35}-s_{13}s_{15}s_{36}+s_{11}s_{35}s_{36}-s_{11}s_{33}s_{56})\}, \end{array}$$

where

3.10 $$M=s_{15}^{2}s_{33}-2s_{13}s_{15}s_{35}+s_{11}s_{35}^{2}+s_{13}^{2}s_{55}-s_{11}s_{33}s_{55}.$$

For simplicity, we have dropped the left-hand side superscript 1 for all components *s_ij_* in equations (3.7)–(3.10). Interestingly, only out of the twin plane shear stresses ($\sigma_{4}^{A}$ and $\sigma_{6}^{A}$) lead to the occurrence of compatibility stresses in type I twinned structures, whereas normal stresses or hydrostatic pressure do not change the internal stress state.

Compatibility stresses in type II twins, by contrast, are a function of all stress components $\sigma_{j}^{A}$; by virtue of equation (2.5) and equations (3.4)–(3.6), the compatibility stresses in variant 1 can be directly calculated from:

3.11 IIσ1C=−2(−1+f)N{[∑i=14(−2fαi+αi+βi+γi)σiA]+(−α5−β5)σ5A+((4f2−4f+1)s15s35s36+(−4f2+4f−1)s16s352+s16s33s55−s13s36s55)σ6A∑i=14},

3.12 IIσ3C=2(−1+f)N{[∑i=14⁡(−2fδi+δi+θi+μi)σiA]+(−δ5−θ5)σ5A+((4f2−4f+1)s152s36+(−4f2+4f−1)s15s16s35+s13s16s55−s11s36s55)σ6A∑i=14}

3.13 $$\begin{array}{rl} \;\text{and}\;{}_{\mathrm{II}}^{}\sigma_{5}^{C} & =\frac{2(-1+f)}{N}\{\left[\sum_{i=1}^{4}(\rho_{i}-\varphi_{i})\sigma_{i}^{A}\right]+((1-2f)s_{15}^{2}s_{33}+(-2+4f)s_{13}s_{15}s_{35}\\ & \;+(1-2f)s_{11}s_{35}^{2})\sigma_{5}^{A}+((1-2f)s_{15}s_{16}s_{33}+(2f-1)s_{13}s_{16}s_{35}\\ & \;+\left(2f-1)s_{13}s_{15}s_{36}+(1-2f)s_{11}s_{35}s_{36})\sigma_{6}^{A}\right\}, \end{array}$$

where we have used the abbreviations

3.14 $$\begin{array}{rl} \alpha_{i} & =-s_{15}s_{33}s_{i5},\;\beta_{i}=s_{13}s_{35}s_{i5}\text{, }\;\gamma_{i}=-2fs_{13}s_{35}s_{i5},\\ \delta_{i} & =-s_{13}s_{15}s_{i5},\;\theta_{i}=s_{11}s_{35}s_{i5}\text{, }\mu_{i}=-2fs_{11}s_{35}s_{i5},\\ \rho_{i} & =s_{13}^{2}s_{i5}\text{, }\;\varphi_{i}=s_{11}s_{33}s_{i5},\\ N & =s_{15}^{2}s_{33}-2s_{13}s_{15}s_{35}+s_{11}s_{35}^{2}-f\zeta +f^{2}\zeta +s_{13}^{2}s_{55}-s_{11}s_{33}s_{55}\\ \;\text{and}\;\zeta & =4s_{15}^{2}s_{33}-8s_{13}s_{15}s_{35}+4s_{11}s_{35}^{2} \end{array}\}.$$

While equations (3.11)–(3.14) contain substantially more terms than equations (3.7)–(3.9), they can be easily evaluated numerically once distinct values can be assigned to the *s_ij_* for a specific material of interest.

To derive analytical solutions for the three compatibility stresses in variant 1 of a compound twin, one can use the additional symmetry condition, equation (2.9), either on equations (3.7)–(3.10) or (less conveniently, but with identical results) on equations (3.11)–(3.13). The compatibility stresses are then given by

3.15 $$\begin{array}{rl} {}_{C}^{}\sigma_{1}^{C} & =2(-1+f)\;\frac{\sigma_{6}^{A}(-s_{16}s_{33}+s_{13}s_{36})}{s_{13}^{2}-s_{11}s_{13}}, \end{array}$$

3.16 $$\begin{array}{rl} {}_{C}^{}\sigma_{3}^{C} & =2(-1+f)\;\frac{\sigma_{6}^{A}(-s_{11}s_{36}+s_{13}s_{16})}{s_{13}^{2}-s_{11}s_{13}} \end{array}$$

3.17 $$\begin{array}{rl} \;\text{and}\;{}_{C}^{}\sigma_{5}^{C} & =2(-1+f)\;\frac{s_{45}\sigma_{4}^{A}}{s_{55}}. \end{array}$$

Despite the high degree of symmetry, compatibility stresses do occur even in compound twins; as is to be expected from equations (3.7)–(3.9), only applied shear stresses $\sigma_{4}^{A}$ and $\sigma_{6}^{A}$ contribute to the compatibility stresses in compound twins because they share the fundamental symmetry properties with type I twins.

For all twin types, compatibility stresses are linear functions of the applied stresses; this relationship can be formally expressed, summarizing the coefficients of the relevant equations in a matrix, *R_ij_*, as

3.18 $${}_{}^{1}\sigma_{i}^{C}={}_{}^{1}R_{ij}^{}\sigma_{j}^{A}.$$

As only $\sigma_{1}^{C}$, $\sigma_{3}^{C}$, $\sigma_{5}^{C}$ are non-zero, only every second row (*i* = 1,3,5) of ^1^*R_ij_* contains non-zero elements; obviously, ^1^*Rij* is not symmetric. By means of equation (3.3), we also find

3.19 $${}_{}^{2}R_{ij}^{}=\frac{-f}{\left(1-f\right)}{}^{1}R_{ij}$$

As shown in the two following sections using specific examples, the compatibility stresses can be of a similar order of magnitude as the applied stresses. The ratio of the volume fractions of variants 1 and 2 considerably affects the absolute values of compatibility stresses: On the one hand, the limiting case of *f* = 1 represents a perfect single crystal without any twin lamellae corresponding to variant 2—consequently, no compatibility stresses occur. On the other hand, very small but non-zero values of *f* can be interpreted as one (or several) thin twin variant(s) 1 embedded in a much larger grain (variant 2). This situation is frequently encountered in materials that deform by mechanical twinning [24] and thus warrants additional analysis. It is obvious from equation (3.3) that for *f* → 0 compatibility stresses in variant 2 (the adjacent grain material surrounding the variant 1 lamella) remain negligibly small, while the compatibility stresses in the thin twin variant 1 can be quite high (but even for *f* → 0 there is a finite upper limit). The physics of the atomic crystal lattice, of course, also put a limit to this scenario: twin variants necessarily have a finite minimum width directly related to the lattice length scale; moreover, twinning requires nucleation and growth (formation of variant 1 in the surrounding lattice of variant 2) which only occurs if a nucleus has a critical size. We do not attempt to study the complex microstructural deformation mechanisms (which in many cases involve elaborate movements and multiplication of partial dislocations) in the present paper, but we highlight that the particularly high compatibility stresses in thin twin variants may considerably affect twin formation and growth both in deformation and transformation twins. The analytical model of compatibility stresses presented here may well contribute to the development of more detailed models in this classical area of materials science research [30,31] that can now also take elastic anisotropy into account.

Using our model of compatibility stresses in twins is simple. The procedure can be summarized as follows: for a given material, the twinning elements (in particular, the orientations of the twin plane and the shear direction), the twin type and the fundamental elastic constants need to be known *a priori*. The elastic compliance matrix (typically defined in a coordinate system based on the symmetry planes of the crystal lattice) then needs to be transformed into the twin coordinate system. For a given state of externally applied stress $\sigma_{j}^{A}$, the compatibility stresses in variant 1 can be directly calculated using equations (3.7)–(3.9), (3.11)–(3.13) or (3.15)–(3.17), for type I, type II or compound twins, respectively. The compatibility stresses in variant 2 can finally be determined using equation (3.3).

In the remaining sections of this paper, we study two examples of the stress states in twinned Cu and in twinned B19′ martensites in the shape memory alloy NiTi to demonstrate the physical significance of compatibility stresses in twinned microstructures. Using these special examples, we also derive additional equations for effective compliance matrices that describe the macroscopic elastic behaviour of materials with twinned microstructures.

## 4. Compatibility stresses and slip system activation in twinned copper

Copper is of key importance in the electronics industry. Driven by a need for increasingly smaller devices and for weight-reduction, growing attention has been focused on high-strength Cu-based alloys with excellent electrical conductivity [32]. Alloying or hardening by cold work can increase the yield strength, but these classical metallurgical methods also increase electrical resistivity. Reducing the grain size by other means and stabilizing the corresponding microstructures by introducing finely spaced nano-scale twins, by contrast, leads to a favourable combination of high strength, substantial work hardening ability and considerable ductility while maintaining excellent electrical properties. Recent ground-breaking research, therefore, has been focused on the finely twinned microstructures and the remarkable properties of electro-deposited (ED) Cu [33]. It is particularly important to obtain a detailed understanding of the multiple interaction processes of dislocations with the nano-scale twin boundaries in ED Cu: twin boundaries can act as effective barriers for dislocation motion, their presence can, for instance, also lead to channelling effects (where dislocation motion is restricted in some and promoted in other directions), or the formation of dislocation pile-ups that create long-range stress fields. Motivated by the dedicated atomistic, micro- and continuum mechanical investigations that have been performed to analyse the relevant microstructural processes [34–36], we consider elastic anisotropy, compatibility stresses and their effect on slip system activation in twinned Cu.

**Figure 5.**
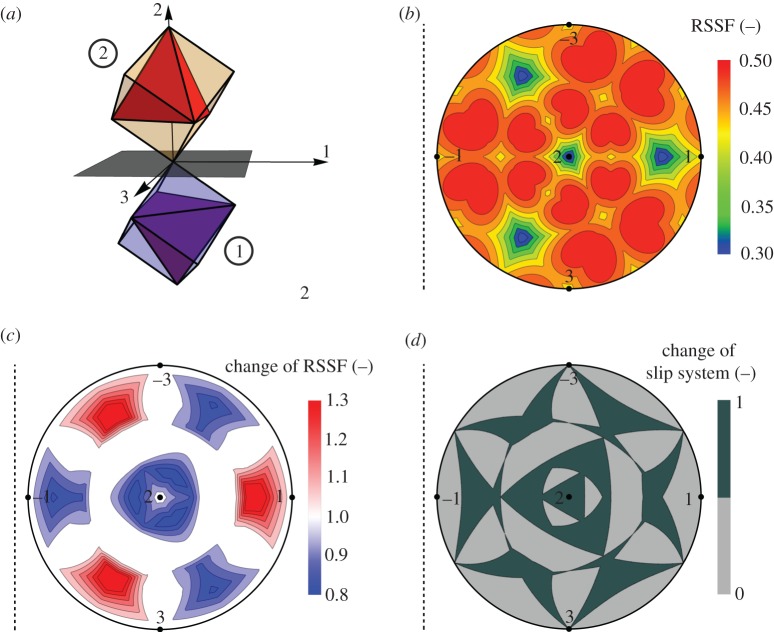
Impact of compatibility stresses on slip system activation in {1 1 1}⟨1 1 0⟩ twins in Cu subjected to uniaxial tensile loading. (*a*) Schematic representation of the cubic unit cells of variants 1 and 2 and of the corresponding slip systems (tetrahedra). (*b*) Maximum RSSF during uniaxial loading of a single crystal with orientation of variant 1. (*c*) Relative change of RSSF values in variant 1 as a function of loading direction when compatibility stresses are also taken into account. (*d*) Analysis of slip system activation in variant 1 including the effect of compatibility stresses. 0: no change of slip system compared with the single crystal; 1: change of slip system. Stereographic plots representing similar datasets for variant 2 can be obtained by reflection of all stereographic plots with respect to the dashed lines indicated in (*b*–*d*).

Compound twins are formed in Cu and in many face-centred cubic (fcc) materials. They are characterized by {1 1 1} twin planes and ⟨1 1 2⟩ twin shear directions. Plastic slip occurs by movement of dislocations with Burgers vectors (i.e. slip directions) of ⟨1 1 0⟩ type and on {1 1 1} slip planes. In this study, we only consider such perfect dislocations, but we emphasize that our model can also be used to investigate the effect of compatibility stresses on the partial dislocations (with ⟨1 1 2⟩ Burgers vectors in fcc materials) that are often formed by dissociation of perfect dislocations in low stacking fault energy materials like Cu. Figure 5*a* schematically shows the orientation of the cubic unit cells of variants 1 and 2 in a {1 1 1}⟨1 1 2⟩ compound twin, highlighting the mirror symmetry between unit cells, the orientation of the twin plane and the twin coordinate system. Moreover, in each unit cell, the four equivalent {1 1 1} type slip planes are indicated as the triangular faces of a regular tetrahedron. In this well-known representation of slip systems in fcc materials [37], the three edges of each face represent the relevant ⟨1 1 0⟩ type Burgers vectors for each slip plane, resulting in a total of 12 slip systems in each variant. Because the twinning plane is also a slip plane, three slip systems are shared by both variants and the corresponding Burgers vectors are contained in the twin plane.

Schmid's Law states that, for a given applied load, a slip system in a single crystal (variant) will be activated once the highest resolved shear stress (i.e. the shear stress acting in the slip plane and in the dislocation slip direction given by the corresponding Burgers vector) reaches a critical value. This is most conveniently expressed in tensor notation [38]:

4.1 $$m_{ij}^{s}\sigma_{ij}=\tau^{s},$$

where $\sigma_{ij}$ is the stress tensor, *m_ij_* relates the Burgers vector *b*^s^ of slip system *s* (*s* = 1, … ,12 in an fcc single crystal), the slip plane normal *n*^s^ and the unit vectors of the coordinate system in which the applied stress tensor is given (here, we choose the twinning coordinate system: *a*^1^ = (1,0,0), *a*^2^ = (0,1,0), *a*^3^ = (0,0,1)):

4.2 $$m_{ij}^{s}=n_{k}^{s}a_{i}^{k}b_{l}^{s}a_{j}^{l}\;(i,j,k,l=1,2,3).$$

To obtain representative information on the effect of compatibility stresses on the activation of individual slip systems in twinned Cu, out of an infinite number of possible applied stress states, we systematically sample the material response to simple uniaxial tensile loading in all possible orientations. This situation corresponds to a tensile test on a specimen taken out of a twinned microstructure with an arbitrary orientation of the load axis. When a unit tensile load is applied in a direction *t* = (*p,q,r*), the stress tensor in the twin coordinate system is given by

4.3 $$\sigma_{ij}^{A}=\left(\begin{array}{ccc} p^{2} & pq & pr\\ pq & q^{2} & qr\\ pr & qr & r^{2} \end{array}\right).$$

To calculate compatibility stresses in the specific case of twinned Cu, we use elastic constants determined experimentally at room temperature [39] (table 1). After transformation of the corresponding compliance matrix into the twin coordinate system, the *s_ij_* values can be used in equations (3.15)–(3.17). Considering the regular structures observed experimentally in ED Cu [36], it is reasonable to assume that *f* = 0.5. The compatibility stresses in variant 1 then are given by

4.4 $$\begin{array}{rl} {}_{}^{1}\sigma_{1}^{C} & =0.597\sigma_{6}^{A}, \end{array}$$

4.5 $$\begin{array}{rl} {}_{}^{1}\sigma_{3}^{C} & =-0.597\sigma_{6}^{A} \end{array}$$

4.6 $$\begin{array}{rl} \;\text{and}\;{}_{}^{1}\sigma_{5}^{C} & =-0.597\sigma_{4}^{A} \end{array}$$

while the compatibility stresses in variant 2 simply change sign.

**Table 1.** Elastic constants of Cu [39] and NiTi B19′ [40] used in this study. Compared with [40], the signs of some *c_ij_* for B19′ NiTi have been adapted to be in line with the labelling of lattice constants as given in the next section. All values have been rounded to 1 GPa. The empty boxes are either zero or given by crystal symmetry.

*c_ij_*/GPa	*c*_11_	*c*_12_	*c*_13_	*c*_15_	*c*_22_	*c*_23_	*c*_25_	*c*_33_	*c*_35_	*c*_44_	*c*_46_	*c*_55_	*c*_66_
Cu	168	121								75			
NiTi–B19′	223	129	99	−27	241	125	9	200	−4	76	4	21	77

As a reference, we first characterize the effect of uniaxial loading on the resolved shear stress in a single crystal (i.e. without additional compatibility stresses) with the orientation of variant 1. Following the original idea of Schmid & Boas [41], we define the ratio of the shear stress in a specific slip system to the unit tensile load as ‘resolved shear stress factor’, RSSF. In the absence of additional stresses, and when only uniaxial loading is applied, the RSSF is also known as Schmid factor and it is well known that it can only have a maximum value of 0.5 (reached in slip planes that are rotated by 45° with respect to the loading direction). In figure 5*b*, the (maximum) RSSF in a single crystal with the orientation of variant 1 is shown for all possible uniaxial loading directions. This graph is produced by means of stereographic projection: the pole of every loading direction is projected from a unit sphere into the equatorial plane and the colour in figure 5*b* maps a specific RSSF value onto the corresponding point. For better orientation, the locations in the stereographic plot associated with loading in the positive and negative directions of the coordinate axes of the twin coordinate system are also indicated in figure 5*b*. Note that an external load usually leads to shear stresses in all possible 12 slip systems. Only the maximum value for each loading direction was used for this plot. Different slip systems are of course activated in different regions of the stereographic plot. The distribution of RSSF values reflects the threefold symmetry that one expects when viewing the fcc crystal along a ⟨1 1 1⟩ direction. The RSSF values vary between a minimum of 0.3 and reach a maximum value of 0.5 in distinct orientations. Four minima of RSSF values occur in loading directions perpendicular to the different {1 1 1} planes. Because of the symmetry of the compound twin, a similar RSSF plot for a single crystal with the orientation of variant 2 can be obtained simply by using the dashed line shown on the left-hand side of figure 5*b* as a mirror.

Equations (4.4)–(4.6) show that the compatibility stresses in twinned Cu can be as high as approximately 60% of the external stresses, and we now discuss how these additional stresses affect and change the activation of individual slip systems in different uniaxial loading directions. For every loading direction, we first calculated $\sigma_{i}^{A}$ using equation (4.3), then we calculated the $\sigma_{i}^{C}$ using equations (4.4)–(4.6), and finally we determined the RSSF resulting from the total stress state (i.e. the superposition of $\sigma_{i}^{A}$ and $\sigma_{i}^{C}$). The stereographic plot in figure 5*c* shows the ratio of RSSF values in the twinned structure (in variant 1) to the RSSF values in the single crystal (data presented in figure 5*b*). Red colouring indicates orientations where higher resolved shear stresses occur in the activated slip system compared with the same orientations in the single crystal case; blue regions highlight orientations where the RSSF values are decreased. Interestingly, RSSF values are locally increased by up to 30%, whereas the most pronounced reduction (in other orientations) is only about 20%. Increasing RSSF values are specifically observed in orientations that, in the single crystal, are associated with the regions of lowest resolved shear stresses (i.e. close to ⟨111⟩ directions; blue regions in figure 5*b*), with the exception of loading directions close to the twin plane normal (2-direction). Similar to figure 5*b*, the stereographic plot of RSSF ratios for variant 2 can be obtained by using the dashed line in figure 5*c* for reflection.

For many loading directions, the additional stresses change which slip system is predominantly activated compared with a single crystal of similar orientation. This effect is clearly highlighted in figure 5*d*; dark colouring indicates orientations where a change of slip system occurs in variant 1 (again, there is a mirror symmetry for the corresponding plot for variant 2). By numerical evaluation using a large number of arbitrarily chosen orientations, we find that about 42% of loading directions are associated with a change of slip system in the twinned microstructure (this percentage does not directly correspond to the area fraction in the stereographic plot because areas from the unit sphere are distorted when projected onto the equatorial plane). Interestingly, a change of slip system does not necessarily occur in orientations where the RSSF values have changed most (see, e.g. orientations close to the 1-direction in figure 5*c*,*d*).

It is obvious from the tetrahedron symmetry of the four relevant slip planes in single crystal Cu that 25% of uniaxial loading directions will activate the three slip systems in a slip plane parallel to the twin boundary (and thus shared by both twin variants). Once compatibility stresses are included in the analysis, however, the fraction of loading directions that activate slip parallel to the twin boundary is increased to 40%. Similar effects of strain localization along distinct channels are often observed in lamellar microstructures. This is typically rationalized by considering the corresponding interfaces as obstacles to dislocations: when the dislocations gliding on slip planes that are inclined to lamellar interfaces are quickly prohibited from further movement, plastic deformation necessarily occurs predominantly on planes that are parallel to the interfaces. Our new results, based on the simple model of compatibility stresses in twins, show that these additional stresses themselves naturally lead to the activation of slip systems with slip planes parallel to the twin boundaries and thus affect the deformation behaviour of ED twinned Cu.

Similar experimental observations have been reported in [36], and were further analysed by large-scale atomistic molecular dynamics simulations (which naturally consider elastic anisotropy of the crystal lattice as a consequence of the phenomenologically defined interaction potentials). In [36], only special load cases (compressive loading perpendicular to, parallel to or at an angle of 45° to the twin boundary) were studied. It was shown that slip is particularly favoured in ‘soft’ directions (i.e. parallel to the twin boundary) when the compression axis is oriented at 45°. Our more complete analysis presented here confirms these earlier results and demonstrates that slip parallel to the twin boundary occurs in all possible loading directions close to 45°.

## 5. Effective macroscopic elastic behaviour of a type II NiTi B19′ martensite twin

In addition to providing information on the internal, elastically anisotropic stress state, our model can also be used to describe the ‘effective’, macroscopic behaviour of materials with twinned microstructures. Since external loading produces, in addition to the elastic strains given by equation (2.2), compatibility stresses that in turn lead to additional straining, the effective elastic behaviour is given by the weighted sum of the total deformation (produced by the superposition of applied and compatibility stresses) of both variants:

5.1 $$\varepsilon_{i}^{\mathrm{eff}}=f\;{}^{1}S_{ij}(\sigma_{j}^{A}+{}_{}^{1}\sigma_{j}^{C})+(1-f)\;{}^{2}S_{ij}(\sigma_{j}^{A}+{}_{}^{2}\sigma_{j}^{C}).$$

By virtue of equations (2.6), (3.18) and (3.19), this expression can also be given as

5.2 $$\varepsilon_{i}^{\mathrm{eff}}=\left[f\;{}^{1}S_{ij}(I_{ij}+{}_{}^{1}R_{ij})+(1-f)K_{ij}\;{}^{1}S_{ij}K_{ij}\left(I_{ij}-\frac{f}{1-f}{}_{}^{1}R_{ij}\right)\right]\sigma_{j}^{A}=S_{ij}^{\mathrm{eff}}\sigma_{j}^{A},$$

where *I_ij_* is the 3 × 3 identity matrix. Equation (5.2) allows to directly calculate the macroscopic elastic deformation of a twinned microstructure for arbitrary applied loads, using only components of the compliance matrix ^1^*S_ij_* of variant 1 and its volume fraction *f* as numerical input.

While it is not directly obvious from the structure of equation (5.2), which contains the non-symmetric ^1^*R_ij_* matrix, a simple argument is sufficient to show that $S_{ij}^{\mathrm{eff}}$ is indeed symmetric: consider the elastic strain energy (density) associated with an externally applied stress state,

5.3 $$U=\frac{1}{2}S_{ij}^{\mathrm{eff}}\sigma_{i}^{A}\sigma_{j}^{A}.$$

Irrespective of the fact that external loading leads to additional internal stresses in twinned microstructures, elastic stresses (and strains) can be superimposed for the simple, linear-elastic material behaviour considered in this paper, and the strain energy of the material subjected to an imposed stress state must not depend on the stress path. Therefore, by virtue of Maxwell's reciprocity theorem

5.4 $$S_{ij}^{\mathrm{eff}}=\frac{\partial^{2}U}{\partial \sigma_{i}^{A}\partial \sigma_{j}^{A}}=\frac{\partial^{2}U}{\partial \sigma_{j}^{A}\partial \sigma_{i}^{A}}=S_{ji}^{\mathrm{eff}}.$$

The inverse of $S_{ij}^{\mathrm{eff}}$, the stiffness matrix $C_{ij}^{\mathrm{eff}}$ is also symmetric. This finding can also be confirmed by using equation (5.2) to compute and compare individual components of $S_{ij}^{\mathrm{eff}}$. This approach can further be used to demonstrate that the corresponding stiffness matrix for twinned microstructures is fully equivalent to results derived in [42] for lamellar materials.

Equations (5.1) and (5.2) clearly show that the occurrence of compatibility stresses directly affects the macroscopic elastic behaviour of twinned materials. As part of the work performed by external loading must be used to generate the additional internal stresses, it is likely that the twinned material appears to be elastically stiffer than an untwinned single crystal of the same material in most load cases. This makes it difficult to experimentally determine the ‘true’ elastic constants of the untwinned lattice for materials that are characterized by twinned microstructures, for instance by resonant ultrasound spectroscopy. One such material, the shape memory alloy NiTi, exhibits special functional properties by virtue of a reversible martensitic phase transformation that allows for high recovery strains of more than 6% [43]. The low temperature phase, martensite, has a monoclinic B19′ crystal structure. Twelve B19′ variants can be formed from the cubic B2 crystal of the high temperature phase, austenite. During the shear-dominated phase transition from austenite to martensite (which can be either thermally driven or stress-induced), nucleation and growth are subject to well-understood constraints at the phase interfaces: there must be a ‘habit plane’ such that both phases fit together without distortion of both lattices. This habit plane condition naturally leads to the formation of twins (by combining two suitable out of the 12 available variants into a so-called correspondent variant pair, CVP; both variants typically need to be present with different volume fractions) in B19′ martensite. The phenomenological theory of martensitic transformations [43,44] accurately predicts the different types of twins (in a total of 192 CVPs) as well as the orientations of habit planes and twin planes that can occur in B19′ NiTi. Most of the predicted type I and type II twins have been observed experimentally. The most frequently observed twin is of type II, with an (irrational) $\left(\bar{0.72}\;1\;1\right)$ twin plane, $\eta_{1}=\{0\;\bar{1}\;1\}$ and *f* = 0.27 [45]. Here we study how the effective elastic properties of martensitic NiTi containing these type II twins are affected by compatibility stresses compared with a theoretical reference state—an untwinned, single B19′ variant. For our analysis, we use the experimentally confirmed lattice parameters *a* = 2.889 Å, *b* = 4.12 Å, *c* = 4.622 Å and a monoclinic angle of *β* = 96.8° [44].

Because of extensive and fine twinning in NiTi, direct experimental measurements of the elastic constants have so far been unsuccessful, and there is an ongoing scientific debate in the shape memory community on how to reasonably define Young's modulus in NiTi martensite [40,46]. Recent *ab initio* calculations, however, provide carefully evaluated estimates of the elastic constants of B19′ ([40], table 1). These data seem to agree very well with various experimental results (e.g. from diffraction studies [47,48]). They are used in the present study as input for equations (3.11)–(3.14), which yield (for variant 1 with *f* = 0.27):

5.5 $$\begin{array}{rl} {}_{}^{1}\sigma_{1}^{C} & =-0.005\sigma_{1}^{A}-0.035\sigma_{2}^{A}+0.034\sigma_{3}^{A}-0.020\sigma_{4}^{A}-0.247\sigma_{5}^{A}-0.382\sigma_{6}^{A}, \end{array}$$

5.6 $$\begin{array}{rl} {}_{}^{1}\sigma_{3}^{C} & =-0.021\sigma_{1}^{A}-0.139\sigma_{2}^{A}+0.135\sigma_{3}^{A}-0.080\sigma_{4}^{A}-0.980\sigma_{5}^{A}-1.852\sigma_{6}^{A} \end{array}$$

5.7 $$\begin{array}{rl} \;\text{and}\;{}_{}^{1}\sigma_{5}^{C} & =0.071\sigma_{1}^{A}+0.471\sigma_{2}^{A}-0.454\sigma_{3}^{A}+0.270\sigma_{4}^{A}+0.129\sigma_{5}^{A}+0.247\sigma_{6}^{A} \end{array}$$

It is worth noting that applied shear stresses $\sigma_{5}^{A}$ and $\sigma_{6}^{A}$ have a strong impact on the local stress state, particularly for ${}_{}^{1}\sigma_{3}^{C}$. In variant 2, because of the higher volume fraction (1 − *f* = 0.73), the (absolute values of) compatibility stresses are smaller by a factor of about 0.37 (note that all numerical values have been rounded to three decimal places):

5.8 $$\begin{array}{rl} {}_{}^{2}\sigma_{1}^{C} & =0.002\sigma_{1}^{A}+0.013\sigma_{2}^{A}-0.013\sigma_{3}^{A}+0.007\sigma_{4}^{A}+0.091\sigma_{5}^{A}+0.142\sigma_{6}^{A}, \end{array}$$

5.9 $$\begin{array}{rl} {}_{}^{2}\sigma_{3}^{C} & =0.008\sigma_{1}^{A}+0.052\sigma_{2}^{A}-0.049\sigma_{3}^{A}+0.029\sigma_{4}^{A}+0.363\sigma_{5}^{A}+0.685\sigma_{6}^{A} \end{array}$$

5.10 $$\begin{array}{rl} \;\text{and}\;{}_{}^{2}\sigma_{5}^{C} & =-0.026\sigma_{1}^{A}-0.174\sigma_{2}^{A}+0.168\sigma_{3}^{A}-0.099\sigma_{4}^{A}-0.048\sigma_{5}^{A}-0.091\sigma_{6}^{A}. \end{array}$$

Since the effective compliance/stiffness matrices for twinned materials are symmetric, twinned microstructures can be considered as triclinic super structures, and 21 independent *s_ij_/c_ij_* components are needed to fully characterize their effective elastic behaviour. To provide a simple analysis of the elastic anisotropy in twinned B19′ NiTi, we again consider uniaxial loading, and we determine the direction-dependent Young's modulus *E_pqr_* for all possible loading directions, given by the unit vector *t*. For triclinic crystal structures, *E_pqr_* is given by [26]

5.11 $$E_{pqr}=\begin{array}{ccccccccccc} p^{4}s_{11} & + & 2p^{2}q^{2}s_{12} & + & 2p^{2}r^{2}s_{13} & + & 2p^{2}qrs_{14} & + & 2p^{3}rs_{15} & + & 2p^{3}qs_{16}\\ & + & q^{4}s_{22} & + & 2q^{2}r^{2}s_{23} & + & 2q^{3}rs_{24} & + & 2pq^{2}rs_{25} & + & 2pq^{3}s_{26}\\ & & & + & r^{4}s_{33} & + & 2qr^{3}s_{25} & + & 2pr^{3}s_{35} & + & 2pqr^{2}s_{36}\\ & & & & & + & q^{2}r^{2}s_{44} & + & 2pqr^{2}s_{45} & + & 2pq^{2}rs_{46}\\ & & & & & & & + & p^{2}r^{2}s_{55} & + & 2p^{2}qrs_{56}\\ & & & & & & & & & + & p^{2}q^{2}s_{66} \end{array}.$$

**Figure 6.**
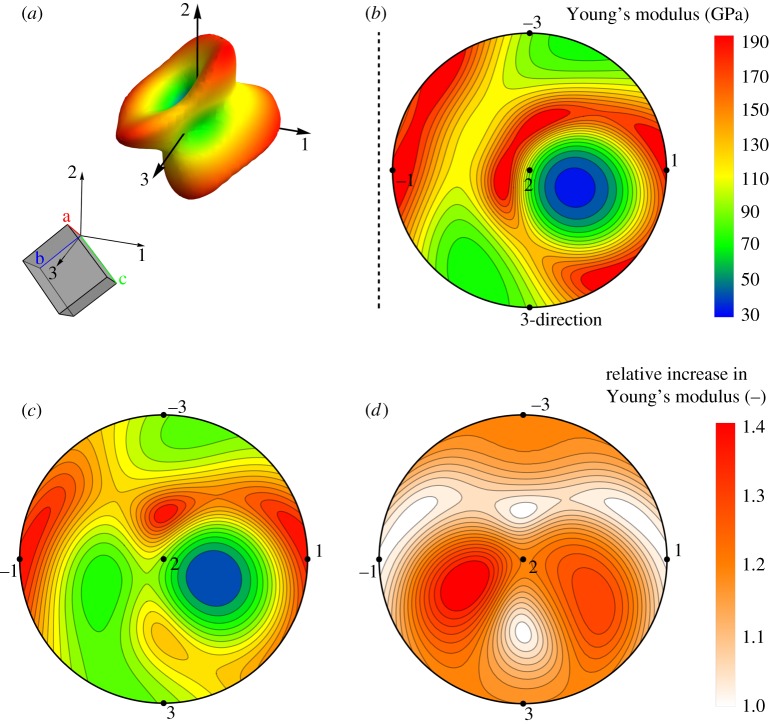
Effective elastic modulus of a type II-1 $\left\{\bar{0.72}\;1\;1\}\;\langle 0\;\bar{1}\;1\right\rangle$ B19′ NiTi martensite twin subjected to uniaxial loading. (*a*) Direction-dependent elastic modulus of variant 1 (orientation of lattice vectors a, b, c relative to the twin coordinate system shown schematically), represented as a three-dimensional surface. (*b*) Stereographic plot of the elastic modulus of variant 2 as a function of loading direction; the dashed line can be used as a mirror line to obtain a similar plot for variant 1. (*c*) The distribution of effective elastic modulus values of the twinned microstructure, but in the absence of compatibility stresses, is dominated by the elastic anisotropy of variant 2. (*d*) When the effect of compatibility stresses is included, the elastic modulus is increased considerably in most loading directions.

It is common to graphically represent *E_pqr_* in three-dimensional plots of a closed surface that provides a clear image of the spatial distribution of *E_pqr_* and its symmetry, which is of course directly related to the symmetry of the underlying crystal structure. Figure 6*a* shows the orientation of the unit cell of variant 1 as well as the corresponding, three-dimensional representation of *E_pqr_*. Because tensile loading occurs simultaneously in positive and negative *t*-direction, the *E_pqr_* surface exhibits an inversion symmetry. For simplicity, we also use stereographic plots for our analysis of the elastic behaviour of single crystalline and twinned B19′ NiTi (figure 6*b*–*d*). Figure 6*b* shows the direction-dependent Young's modulus of the dominant variant 2 (with a volume fraction of 1 − *f* = 0.73; the dashed line in figure 6*b* can again be used for a mirror operation to obtain a similar plot for variant 1, which directly corresponds to the *E_pqr_* surface shown in figure 6*a*). Because of the orientation of the B19′ unit cell with respect to the twin coordinate system, the twofold symmetry of monoclinic crystal structures is obscured in the elastic response and the entire stereographic plot area is needed to represent *E_pqr_*. As discussed in [40], a single B19′ variant is characterized by maximum/minimum *E_pqr_* values of 189.8 GPA/33.2 GPa, respectively, which corresponds to an anisotropy factor of about 5.7. An average elastic modulus can be estimated by Voigt–Reuss–Hill averaging [49] as 122.3 GPa [40], while numerical averaging performed in the present study by evaluating data from 60 000 arbitrary orientations leads to an average elastic modulus of 114.4 GPa.

In a first, naive estimate of the macroscopic elastic behaviour of the NiTi B19′ type II twin, one can neglect the effect of compatibility stresses by simply dropping the two terms with matrices ^1^*R_ij_* from equation (5.2). The predicted elastic properties of the twinned structure then correspond to a linear combination of the elastic properties of variants 1 and 2 (using their volume fractions as weights, figure 6*c*). In this scenario, which does not include the effect of compatibility stresses, the material behaviour is clearly dominated by the properties of variant 2 because of its larger volume fraction (as evidenced, for instance, by the locations of minimum and maximum *E_pqr_* values). Figure 6*c* also shows that in many orientations, the elastic modulus values of variant 2 are reduced in the twinned structure and that the overall anisotropy ratio is thus slightly decreased. Obviously, by arranging variants 1 and 2 according to their twin symmetry relation, and by removing all relevant constraints at the twin boundary, elastically stiff orientations in one B19′ variant are frequently counter-balanced by elastically more compliant orientations in the other variant. The numerical average elastic modulus is reduced to about 106.7 GPa (similarly, the Voigt–Reuss–Hill average, determined from equation (5.2) and also neglecting the ^1^*R_ij_* terms, is reduced to 112.4 GPa).

While the simple averaging procedure used to obtain the data (presented in figure 6*c*) seems to indicate that twinned B19′ NiTi effectively becomes elastically more compliant compared with a B19′ single crystal (figure 6*b*), this conclusion is actually misleading. When compatibility stresses are included in the analysis (by use of equation (5.2) with the components of ^1^*R_ij_* as given in equations (5.5)–(5.7)), the material's response is changed considerably. In figure 6*d*, we show the relative change of direction-dependent Young's modulus values in the twinned structure, subjected to external loading (as well as the corresponding compatibility stresses), compared with the simple average reference case (figure 6*c*) that neglects the effect of compatibility stresses. Figure 6*d* shows that the effective elastic modulus of type II twinned B19′ NiTi martensite is increased by up to 40% in distinct orientations, most notably in orientations close to compliant orientations of variant 1. Indeed, in almost all possible loading directions, the effective elastic modulus is markedly increased. While it remains nearly constant only in very few directions (for instance, in the light-coloured band spanning from the positive to the negative 1-direction in figure 6*d*, the increase is less than 0.05), we observe no case where the occurrence of compatibility stresses leads to a reduction of the effective elastic modulus. We assume that distinct orientations can in principle be present where the elastic modulus is not affected by compatibility stresses either because the three stress components vanish simultaneously or because the additional strains in loading direction in variants 1 and 2 cancel each other out macroscopically. The pronounced impact of compatibility stresses is also confirmed by considering the average elastic modulus values: With 120.8 GPa (numerical averaging), and 124.7 GPa (Voigt–Reuss–Hill average), respectively, we observe an increase compared with the twinned reference case up to values that even slightly exceed the average elastic moduli of the single crystal B19′ case [40]. Further work is required to fully analyse the elastically anisotropic behaviour of the many different types of B19′ twins in NiTi. The first results presented here, used merely as an example case, clearly indicate that the effective material response of twinned structures is affected by compatibility stresses, and that neglecting their effect can lead to erroneous conclusions when assessing local and macroscopic stress states.

## 6. Summary and conclusion

In this paper, we have developed a simple yet very general model of elastic compatibility stresses in twinned microstructures. These stresses must occur at twin boundaries to provide compatibility at the plane interfaces between two neighbouring twin variants. Our model makes use of the special symmetry of twinned microstructures, building on the fact that only a few components of the stiffness (and compliance) matrices of both twin variants exhibit a change of sign when described in the twin coordinate system, which is tied to the twinning elements, shear direction and twin plane normal. The analytical equations derived for type I, type II and compound twins allow to directly calculate the total stress state for arbitrary applied loads. The only input needed to perform such calculations are (i) the lattice constants (i.e. the geometry of the crystal structure) of the material under consideration, (ii) its elastic constants, and (iii) the twinning elements. Stresses and strains are conveniently calculated in the twin coordinate system. Compatibility stresses are constant throughout each variant and can be of the same order of magnitude as the applied stresses, or even higher—particularly in variants with a lower volume fraction.

In addition, we have briefly considered two relevant materials science examples for metallic materials that are strongly affected by their twinned microstructures, electro-deposited Cu and martensitic B19′ NiTi. Using our simple model, we have shown that compatibility stresses can have a considerable effect on slip system activation in twins. We suspect this mechanism to have far-reaching consequences for the analysis of plastic deformation in many metallic materials because the local stress state (which needs to include the compatibility stresses) may well play a crucial role in determining the interaction of dislocations with twin boundaries, by promoting dislocation movement parallel to the twin boundaries, or by influencing twin nucleation and growth processes. To describe their macroscopic behaviour, twinned structures can be considered as triclinic super-structures. Because of the occurrence of (internal) compatibility stresses during external loading, twinned materials appear to be elastically stiffer than similarly oriented single crystals in most load cases.

We close with brief notes on scaling effects and local plastic deformation. Our simple model is based on continuum mechanics and, therefore, it is scale-invariant. Additional scaling effects, such as for instance (grain size) effects on plastic deformation in twinned Cu, are not included. Only the ratio of volume fractions (which in the model corresponds to the ratio of variant lamella widths), *f*/(1 − *f*), plays a part in determining the compatibility stresses, but absolute twin width does not. The model can, therefore, be applied for an analysis of materials with large twin widths of several tens or hundreds of micrometres (as observed in large annealing twins in coarse-grained metals), and it is equally valid in the case of materials with nano-scaled twins, where twin widths often are only small integer multiples of the lattice constant. It is well known from atomistic simulations that even in exceedingly small volumes, stiffness matrices can be used to describe the elastic anisotropy of crystals. Our model, therefore, allows to calculate compatibility stresses in fine twins as long as the elastic constants do not change because of size effects. This certainly happens in the limiting case where monolayers of atoms form each variant. Then, every atom is part of an interface and there may be a need to define different elastic constants in the framework of our model.

Interfacial defects need not be present in twin boundaries when *K*_1_ is a rational lattice plane (i.e. in type I and compound twins) and are thus irrelevant with respect to compatibility stresses. In type II twins, however, the interaction of anisotropic stress fields of interfacial defects, like steps or ledges, with compatibility stresses may open up an interesting field of research. Finally, one way to reduce compatibility stresses is, of course, the local onset of plastic deformation by dislocation slip. By introducing additional dislocations in the vicinity of, or into, a twin boundary, the local geometrical constraints can be relaxed and compatibility stresses can be partially reduced even in type I or compound twins. As discussed in [23] for the bicrystal case, the effect of (reasonably small amounts of) plastic deformation can in principle be included in the framework of our model. The predictions of the simple elastic model presented here, therefore, provide accurate results prior to the onset of plastic deformation, and a reasonable estimate of the upper limit of compatibility stresses thereafter.

## Data accessibility

This article has no additional data.
